# Supplementary material for: The psychosocial burden of cutaneous leishmaniasis in rural Sri Lanka: A multi-method qualitative study
Source: PLoS Negl Trop Dis. 2024 Jan 18;18(1):e0011909. doi: 10.1371/journal.pntd.0011909 (PMC10826957; doi:10.1371/journal.pntd.0011909)
Supplement: S1 Text — (PDF) [file pntd.0011909.s001.pdf]

Unearthing the untold story of the sand fly's disease

Empowering people with cutaneous leishmaniasis: intervention programme to improve patient journey and reduce stigma via community education

Participant details

Your Name - ……………………………………………….
Sex - ……………………………………………….
Age - ……………………………………………….
Your Address -……………………………………………….
Village - ……………………………………………….
Grama Niladhari Division -…………………………………….
MOH division - ……………………………………………….
Telephone Number -……………………………………….

The date you received the booklet - ……………………
The date you handover the booklet - ……………………

Details of the researcher - ……………………
Name of the researcher - ……………………
Telephone number - ……………………

Leishmaniasis related details

Date you first noticed the symptoms of Leishmaniasis-
………………………………………………………………

Date of the confirmation of the disease
………………………………………………………………

Current status

- ☐ Cured
- ☐ On Treatment
- ☐ Treatment discontinued

Purpose

We face many health issues in day-to-day life. When we get sick, it affects not only us but also the loved ones of our life. Leishmaniasis or what is commonly known as “the sand fly’s disease” is one of the many health issues we face. Even though we would love to come and meet you personally and talk with you about your experiences with the disease, Covid 19 has paused a great barrier between us and you. Therefore, we prepared this small booklet to gather knowledge from you and get to know your experience so that after the situation gets better, we can come and meet you in person. We are planning on using the knowledge generated through this book to improve and adjust our project to be more fitting and context-specific that matches your community.

Through this booklet, we are planning to gather knowledge on,

1. Your experiences, the barriers you faced, your journey with the disease, people who supported you since the day noticed the symptoms and until you finished treatments

2. The emotional and social challenges you faced, stories, and beliefs you heard regarding the disease

So how should you write this book?

For your convenience, we have added 11 questions to this book. Each question has 3 pages to answer. Before you start answering we invite you to read the questions first. And don’t forget to answer all the questions! When you are writing the answer, please write it in any way that feels comfortable to you. If you write the answers in a detailed elaborated way, it is easier for us to understand your story. Don’t leave out small details thinking it’s irrelevant. Everything you write is relevant and important to us. So, write anything and everything that comes to your mind.

Thank you so much for joining our study by taking the time and writing this book.

Q1. Do you remember the day that you first noticed the lesion? What happened on that day? Can you explain to us the things that you did first?

Q2. What did you feel when you first noticed the lesion? What did you feel when you got to know that you have got the sand fly's disease ('*wali massage lede',* the local term for leishmaniasis)?

Q3. Did you think that this condition should be medically treated? Was there someone who persuaded you to get treatments? Can you explain the things that you did, the places that you visited, the people that you met, and the type of treatments that you underwent in order to treat this disease? (If you tried any home remedies, it is really important to mention that as well)

Q4. What were the responses that you received from,

I. Your community/society
II. Your family
III. Your friends since you got the Sand fly's disease?

We are keen to hear about both positive and negative responses that you experienced and felt.

Q5. What are the incidents you went through (situations where either you felt relief or discomfort) when you visited different places and you met certain people during the journey of seeking treatments for this disease? (You can make your explanation in relation to the following places that you may have visited)

I. Government hospital
II. Private medical center
III. Traditional or Ayurvedic medicine
IV. Other place or any other person that you met

Q6. What are the physical, mental and social changes that happened in your life after you got this disease? If such kind of change took place, as you feel, can you tell us why did that happen? (Think about both positive and negative changes when you explain)

Q7. It is not so easy to visit the clinic while attending to your day-to-day activities. Therefore, we would like to hear about the difficulties that you faced when you attended the clinic. So tell us, on the first clinic day, how did you feel? What did you feel about the clinic? Did you attend the clinic happily? Or else what were the difficulties that you faced during the clinic?

Q8. When you visit the clinic, you may have come across people with this disease. You may have talked with them. Is there any person(s) among that patients' group that you remember especially? Can you explain to us, what did you feel about that person(S)? What is their opinion about the disease? Have they ever talked with you about any incident where they have felt or faced any negative reactions or discrimination from society because they are having this disease? If you can explain in detail, it'll be easier for us to fully understand the situation.

Q9. We would like to know about the general opinion and thoughts on the Sand fly's disease. In general, how this disease is called in your community, how does the community thinks this disease is caused, how severe and important do they think this disease is? What are the community opinions about the disease? You can write anything here. We are trying to understand, your perception of what kind of disease is this?

Q10.Can you simply draw/illustrate the journey of treatment-seeking from the date that you noticed the lesion up to the end of treatments? (In the illustration, include the incidents that happened, people that helped you, situations where you were discouraged). A model diagram is presented below. There are no rules that your diagram has to be in the same way that we have presented here. You can draw your journey in your own way.

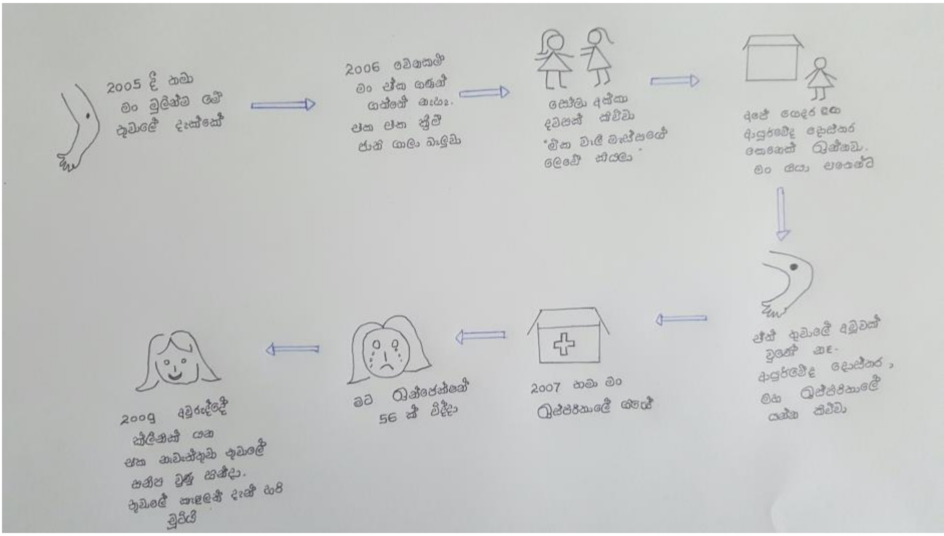

Q11.You may have come across many people and faced incidents that really encouraged you as well as discourage you. You can write about them below.

People and incidents that encouraged me….

People and incidents that discouraged me….
