# Supplementary material for: The psychosocial burden of cutaneous leishmaniasis in rural Sri Lanka: A multi-method qualitative study
Source: PLoS Negl Trop Dis. 2024 Jan 18;18(1):e0011909. doi: 10.1371/journal.pntd.0011909 (PMC10826957; doi:10.1371/journal.pntd.0011909)
Supplement: S1 Table — (DOCX) [file pntd.0011909.s002.docx]

Socio-demographic and CL lesion characteristics of PERJ participants

| ID | Sex | Age (Years) | Occupation | Location of the lesion(s) | Nature of the lesion |
| --- | --- | --- | --- | --- | --- |
| J01 | Female | 55 | Farmer | Leg | Ulcerated wound |
| J02 | Female | 27 | Daily wage labourer | Leg | Papule |
| J03* | Male | 24 | Helper in a bakery | Hand | N/A |
| J04 | Male | 74 | Retired bank worker | Leg | Wound |
| J05 | Male | 43 | Salesman | Leg | Wound |
| J06 | Female | 31 | Daily wage labourer | Leg | Macule |
| J07 | Female | 32 | Self-employed | Below eye | Papule |
| J08 | Male | 26 | Family business | Ear | Nodule |
| J09 | Male | 36 | Masonry | Hand | Papule |
| J10 | Female | 41 | Unemployed | Leg | Small wound |
| J11* | Male | 71 | Farmer | Hand | N/A |
| J12 | Male | 45 | Farmer/Photographer | Abdomen | Papule |
| J13* | Female | 75 | Unemployed | Hand, Leg | Not done |
| J14 | Male | 56 | Farmer | Leg | Wound |
| J15 | Female | 54 | Unemployed | Finger | Ulcerated wound |
| J16 | Male | 39 | Driver | Hand | Wound |
| J17 | Female | 66 | Unemployed | Leg | Wound |
| J18 | Male | 73 | Unemployed | Hand | Nodule |
| J19 | Male | 44 | Carpenter/Farmer | Hand, Leg | Wound |
| J20 | Male | 32 | Management Assistant | Forehead | Papule |
| J21 | Male | 53 | Daily wage labourer | Ear | Papule |
| J22* | Female | 18 | Unemployed | Leg | N/A |
| J23 | Male | 61 | Farmer | Hand | Wound |
| J24 | Female | 72 | Unemployed | Leg | Wound |
| J25 | Male | 65 | Farmer | Hand | Wound |
| J26* | Male | 32 | Management Assistant | Hand | N/A |
| J27 | Female | 61 | Farmer | Nose | Nodule |
| J28 | Male | 45 | Farmer | Below eye, Back, Eye | Wound |
| J29 | Male | 50 | Retired army officer | Leg | Wound |
| J30 | Female | 47 | School teacher | Leg | Wound |

*PERJ interview was not conducted; N/A

|  | Nachchaduwa |  | Thalawa |  | Padaviya |
| --- | --- | --- | --- | --- | --- |
