# Supplementary material for: The psychosocial burden of cutaneous leishmaniasis in rural Sri Lanka: A multi-method qualitative study
Source: PLoS Negl Trop Dis. 2024 Jan 18;18(1):e0011909. doi: 10.1371/journal.pntd.0011909 (PMC10826957; doi:10.1371/journal.pntd.0011909)
Supplement: S2 Table — (DOCX) [file pntd.0011909.s003.docx]

| ID | Sex | Age  (Years) | Occupation |
| --- | --- | --- | --- |
| D01* | Female | 45 | Daily wage labourer |
| D02 | Female | 31 | Daily wage labourer |
| D03 | Female | 27 | Daily wage labourer |
| D04 | Female | 50 | Kindergarten teacher |
| D05 | Female | 55 | Self-employed |
| D06 | Male | 55 | Retired army officer |
| D07 | Male | 24 | Daily wage labourer/Traditional dancer |
| D08** | Male | 50 | Government official |
| D09 | Female | 24 | Housewife |
| D10 | Female | 21 | Student |
| D11 | Male | 28 | Development officer |
| D12 | Male | 63 | Retired school teacher |
| D13** | Female | 43 | Housewife |
| D14 | Male | 71 | Farmer |
| D15 | Female | 28 | Development officer |
| D16 | Female | 71 | Housewife |
| D17 | Female | 66 | Traditional healer |
| D18 | Female | 39 | Economic development officer |
| D19 | Male | 48 | Monk |
| D20 | Male | 50 | School teacher |
| D21 | Female | 50 | Retired Army officer |
| D22 | Male | 70 | Farmer |
| D23 | Male | 65 | Famer |
| D24 | Female | 19 | Student |
| D25 | Male | 54 | Monk |
| D26 | Female | 54 | Famer |
| D27 | Male | 51 | Retired army officer |
| D28 | Female | 52 | Farmer |
| D29* | Female | 27 | Management trainee |
| D30 | Female | 26 | Student |

Details of the auto-ethnographic diary study participants

*The diary was not returned

**The follow-up diary interviews were not complete

|  | Nachchaduwa |  | Thalawa |  | Padaviya |
| --- | --- | --- | --- | --- | --- |
