# Supplementary material for: The psychosocial burden of cutaneous leishmaniasis in rural Sri Lanka: A multi-method qualitative study
Source: PLoS Negl Trop Dis. 2024 Jan 18;18(1):e0011909. doi: 10.1371/journal.pntd.0011909 (PMC10826957; doi:10.1371/journal.pntd.0011909)
Supplement: Attachment — Submitted filename: Response to the reviewers.docx [file pntd.0011909.s004.docx]

**Response to the reviewers**

We thank all the reviewers for their constructive comments. The responses to all comments are listed below.

| Reviewer #1 Comments | |
| --- | --- |
| Methods | |
| The purpose of the study, as described by the authors, is to explore the psychosocial burden of cutaneous leishmaniasis in rural Sri Lanka through a multi-method qualitative approach. The study design is appropriate for the stated objectives since the authors employed a multi-method qualitative approach that gathered data on the psychosocial burden of cutaneous leishmaniasis using in-depth interviews, focus group discussions, and participant observation. The study aims to investigate the correlation between cutaneous leishmaniasis and rural populations of Sri Lanka. The research design is justified by the authors as rural communities in the area are more prone to the disease and face particular psychosocial challenges. Although the sample size is not stated, the authors state that they used purposive sampling methods to select study participants. They assert that this method enabled them to choose participants who were most likely to offer comprehensive and varied data on the psychological and social impact of cutaneous leishmaniasis. The researchers examined the collected data via qualitative analysis techniques which included thematic analysis. They maintain that these techniques were fitting for the gathered information and facilitated the identification of crucial themes associated with the psychosocial burden of cutaneous leishmaniasis in the rural areas of Sri Lanka. The study received ethical clearance from the Ethics Review Committee of the Faculty of Medicine & Allied Sciences, Rajarata University of Sri Lanka, and the Faculty of Medicine and Health Sciences, Keele University, United Kingdom. The authors ensured informed consent, confidentiality, and privacy for study participants. | We thank the reviewer for this comment |
| Results | |
| The authors employed qualitative data analysis methods, specifically thematic analysis and constant comparison, to analyze the data collected during the study. They use quotes from study participants to highlight the critical themes associated with the psychosocial burden of cutaneous leishmaniasis in rural Sri Lanka.  The provided visuals effectively convey the information required to comprehend the study. | We thank the reviewer for this comment |
| Conclusions | |
| The study data supports the authors' conclusions. They utilize participant quotes to emphasize critical psychosocial themes related to cutaneous leishmaniasis burden in rural Sri Lanka, highlighting the significance of their findings for public health interventions and disease policies. Discuss the limitations of their analysis, including potential bias in the data collection process, possible reluctance of some participants in sharing their experiences, and the fact that the study was conducted in a specific geographic location that may limit its generalizability to other settings. Nevertheless, highlight the helpfulness of their data in advancing our understanding of the psychosocial burden of cutaneous leishmaniasis in rural Sri Lanka. Argue that their findings have significant implications for the development of public health interventions and policies pertaining to the disease. They propose that their research emphasizes the necessity of more detailed and integrated methodologies in handling the psychosocial weight of cutaneous leishmaniasis. These methodologies involve improving healthcare accessibility, reducing stigma, and raising community awareness about the disease.  Address the public health relevance of their study by stating that cutaneous leishmaniasis is a neglected tropical disease that disproportionately affects rural communities in developing countries. It can have significant psychosocial impacts on affected individuals and their families. The study underscores the need for more research and interventions that can address the psychosocial burden of the disease and improve the lives of affected individuals. | We thank the reviewer for this comment |
| In brief, this article presents findings from a study examining the psychosocial impact of cutaneous leishmaniasis on individuals and families in rural Sri Lanka. The multi-method qualitative approach included in-depth interviews, focus group discussions, and participant observation to gather data. The authors focus on the disease's psychosocial burden and its implications for those afflicted and their families. The article follows conventions in structure, author and organization formatting, and citation style. Objectivity is maintained through the use of plain, concise language, passive tense, and impersonal construction. The writing is jargon-free, formal, and free of filler words and figurative language. The article maintains a balanced tone and avoids subjective judgments. Grammatical accuracy, punctuation, and spelling are consistent. They analyzed the data by using qualitative data analysis methods and found various key themes associated with the psychosocial burden of cutaneous leishmaniasis, such as stigma, fear, and social isolation. The authors assert that their conclusions imply significant consequences for public health interventions and policies concerning the disease and emphasize the requirement for more inclusive and consolidated approaches to cope with the psychosocial burden of cutaneous leishmaniasis. Overall, the study provides a detailed and informative account of its findings. The authors present their data clearly and comprehensively and offer a thoughtful discussion of the implications for public health policies and interventions. Nonetheless, it is a valuable contribution to the literature on the psychosocial burden of cutaneous leishmaniasis, highlighting the need for further research and interventions to address this neglected tropical disease. | We thank the reviewer for this comment |

| Reviewer #2 Comments | |
| --- | --- |
| 1 Methodology Clarifications | |
| 1-1 The manuscript begins with the ECLIPSE methodology, which was later complemented by the auto-ethnographic diary study due to COVID-19 challenges. It would be beneficial to clarify the transition between these methods and their respective contributions to the study's results | We thank the reviewer for this comment. In response, we have revised the methodology section to clarify that the "ECLIPSE methodology" was not a distinct methodology but rather the approach we employed for this study. Additionally, to provide further clarifications on the modifications done because of COVID-19, we have included a citation to our paper in which our team documented the transition process. (Lines 120-123) |
| 1-2 The title suggests a multi-method qualitative study, but the methodologies used seem more like variations of a qualitative approach. Please consider revising the title for accuracy. | Ethnographic research is always a multimethod qualitative study [1–3]. The approach for the participant observation and diary study is different from the approach for the PERJ where our intention was to explore the experiences of people with CL as well as on how they feel about the experiences (reflections). Therefore, we think its opportune to keep the original title.   1. O’reilly K. Ethnographic Methods -The practice of ethnography. 2019. Available: <http://www.routledge.com/books/details/9780415561815/.MetadataRecord:https://dspace.lboro.ac.uk/2134/15689> 2. Herbert S. For ethnography. Prog Hum Geogr. 2000;24: 550–568. 3. Mack N, Woodsong C. Qualitative research methods. 2005. |
| 2 Participant Selection: | |
| Lines 193-195 detail participant recruitment, but earlier sections (Lines 127-175) focus heavily on the ECLIPSE method. It would enhance clarity if the manuscript concentrated more on the selection process for the current study's interviewees. | Thank you for this comment. We have made revisions to reporting the methods of our study, (Lines 109-127) and we revised the entire methods section (highlighted). These changes should now clearly convey that ECLIPSE is the overarching program within which the Sri Lankan specific study was conducted. |
| 3 Ethical Considerations: | |
| Were there any ethical considerations taken into account when modifying the study methodology during the COVID-19 pandemic? Did these changes adhere to the initial protocol, or were they self-decisions by the authors? | We have obtained ethical approval from both Sri Lanka and the UK, and we have now duly cited this approval in the paper to provide clarity (Line 269). To summarize,  We adhered to the initial protocol when changes were made in the methods. The initial protocol allowed researchers to apply/choose a number of qualitative methods which would fit the contexts studied no major ethical deviations were present. Infact the methods were changed due to ethical reasons as the communities pointed out that participant observation would not be a suitable method concerning the safety of the community members as well as the researcher. There was stigma related to acquiring COVID-19 at the initial stage and an official from outside meeting community members created an unfavourable situation to the particular community members within the village. Further we adhered to all possible ethical procedures while using the new methods such as informed written consent, safeguarding privacy and confidentiality in transcribing and using the excerpts (using pseudonyms at all instances) and data storage. |
| 4 Data Analysis: | |
| 4-1 The sources of the thematic analysis should be specified, differentiating between individual interviews and additional document analyses. | Thank you for this comment. All data collected whether its diary or interview transcripts form one big data source. We have now clearly mentioned how the different sources of data were coded. This was corrected (Line 250-258) |
| 4-2 How was the analysis software used, and how were nodes for Figure 3 created? | As its conventional in qualitative research thematic analysis is performed on data (ethnographic field notes, interview transcripts, written diaries etc). We have not used any software because it does not support the native language (Sinhala) and in contrast to quantitative data analysis software (Strata, SPSS), qualitative software like NVivo is used only for data storage and qualitative researcher has to manually conduct the data analysis (coding).  We used Excel tables to place the codes/nodes in rows and place each participant information in columns so that the excerpts of data from each source were clearly placed according to the nodes to compile a master data sheet in which the patterns and associations are easy to detect and formulate into subthemes and major themes. The nodes in the figure were based on this analysis outcomes (subthemes and codes). Due to the manuscript's word count limitations, we are unable to provide a comprehensive explanation of all the details within it. |
| 5- Discussion and Conclusion Refinement: | |
| Both sections should more accurately reflect the primary analysis derived exclusively from the interviews. | Post-PERJ interviews constituted just one of the four data components utilized in our analysis. The ultimate findings and outcomes of our study were derived from a comprehensive analysis of all four data sets, (lines 247-251). To enhance transparency regarding our data analysis, we have incorporated excerpts from field notes, (line 513-526), PERJ (line 376-379) and post-PERJ interviews (line 391-396) within the text and we have revised the verbatims to indicate whether it is from a PERJ or a post-PERJ interview (as PERJ or PERJ-i) (Line 297-298). We would like to emphasize that post-PERJ interviews were never treated as standalone data in any phase of our research. As per comments above, the overall data set is large and robust one for ethnographic research studies and we are pleased to report that we included all four data sets. |
| 6- Limitations about the insider Perspective: The authors' prior knowledge from the ethnographic study might have influenced the thematic analysis. It would be insightful if the interview results were analysed independently by a researcher not involved in the initial study. | The main goal of conducting any ethnographic study is to conduct reflexive fieldwork, which, indeed includes the perspectives of researchers and to gain the insider perspective of the studied context^1^ (Line 145-146). Analyzing the data without the contextual understanding of culture, beliefs, and the significance behind expressions, as obtained through the ethnographic study, negates the initial intent of including an ethnographic component in our research. Hence the influence of the researchers who conducted the ethnographic study in the data analysis process is an advantage and ethical as it preserves the true meanings of the expressions of participants, as they had the knowledge of the meanings behind expressions and the cultural contexts in which the study was conducted. Nevertheless, two investigators independently coded the transcripts and consensus on the meanings, subthemes and themes were taken after discussing (250-252). We believe this process was reflexive and would have avoided any biases that could have been introduced by analysis of a single researcher.  ^1^Charmaz K. Constructing Grounded Theory. SAGE publications; 2006 |
| Minor Comments: | |
| 7 Literature Review: | |
| Lines 93-95: Suggest revisiting the claim about the "neglected" area of psychosocial implications of CL. Some existing research in this field should be acknowledged. | This has been revised to include additional information about the existing studies, as pointed out by the reviewer. line 85-89 |
| 8 Demographic Details: | |
| Lines 115-116: Please provide context on the relevance of the demographic details (Sinhalese ethnicity and Buddhist population) to the study's objectives. | The demographic details are provided to offer potential readers insights into the community context and to maintain transparency regarding the contexts in which the study was conducted. Specifically, it would justify the ethnicity of the study participants and help explain data analysis steps conducted in the native language. |
| 9 Figures and Time Frames: | |
| 9-1 For Figure 2: It would be beneficial to include the country and district locations, along with the scale. | This was corrected (Figure 2) |
| 9-2 Clarify the time frame for the selection of the three GN divisions with the highest CL prevalence. | This is corrected (Line 140) |
| 10- Results Presentation: | |
| 10-1 The gender ratio reported doesn't seem to reflect the epidemiological trend of CL in Sri Lanka. Was this intentional, and if so, why? | Although the reported gender ratio is slightly favoured towards male (which probably might be due to the epidemics among the male army personnel during the period of humanitarian conflict), our aim was to explore the psychosocial burden among different types of affected individuals. Therefore, instead of strictly adhering to the epidemiological trend, our primary objective was to maximize diversity in terms of age, lesion site, and occupation among our study participants. As outlined in the methodology section, we used snowballing with the use of CEI to identify and purposefully recruit participants (line 220-221). We believe that this strategy has generated credible data representing a range of different participants. |
| 10-2 Age ranges should be reported by gender in the main results section. | This was corrected Line 279-281 |
| 10-3 Clarify which pieces of information were inductively derived and which were deductively inferred. Within the transcript or other sources analysed. | We have used is inductive analysis as we have specified in (line 258-259). All information is inductively derived. |
| 11- Method Section Refinement: Consider streamlining the method section to focus primarily on the 30 participant interviews, rather than extensively detailing the ECLIPSE study. | Thank you, reviewer, for this comment. We have made revisions to the methods section to clearly indicate that this study was conducted as part of the ECLIPSE project, signifying that both studies share the same methodology (Highlighted). We would like to again clarify that this study has four data sets all of which have been analysed comprehensively were used to inform the results presented here. |
| 12- References: Please check the reference list for repetitions and inconsistencies, | Thank you reviewer for pointing this out. This was corrected. |

| Reviewer #3 Comments | |
| --- | --- |
| Introduction | |
| Page 3, line 45: “a diverse an array..”  - please remove “an” | This is corrected line 45 |
| Page 3, lines 57-58: “Prioritising the psychosocial burden..” – The psychosocial burden of CL or Leishmaniasis in general? | This is corrected line 57 |
| Page 4, line 78: “impact an individual’s socioeconomic status..” – impact on an or on the individual’s socioeconomic status? | This is corrected line 77 |
| Page 4, line 82: “leaving out the other complex psychosocial burden attached..” – leaving out other complex psychosocial burdens? | This is corrected line 81 |
| Page 4, line 86: “Research showed that the..” – Suggest rewording as “previous research has shown that..” | This is corrected line 85 |
| Page 4, line 89: If leishmaniasis was added to the public health surveillance system again in 2008, when was it previously notifiable? | In response to the reviewer's comment, we would like to highlight that, as referenced in our manuscript, recent evidence has emerged indicating that CL has been present in the country since 1904, contradictory to what was previous believed. Following the colonial era, it wasn't until 2008 that CL was officially declared as a notifiable disease. To address this, we have made modifications to lines 108-110 in the manuscript to accurately reflect this historical context. |
| Page 5, line 96: among “the” neglected tropical diseases | This sentence was removed |
| Methods | |
| 9.Page 6, line 119: “highest CL cases in Sri Lanka” – Please consider using either highest CL prevalence or highest CL burden. Also, is there a reason why the disease burden is one of the highest in this district? That information might be important for the reader. | This is corrected line 135. |
| 10. Page 6, line 120: It is important to clarify that GNs are subdivisions of the divisional secretariats if I understood correctly. | This is corrected line 137 |
| 11.Page 7, line 129: Please clarify that “health and disease” refer to CL here | This is corrected line 146. We would like to highlight that in here we meant beliefs, local perceptions and practice related to general health not merely focusing on CL as this was the ethnographic study. |
| 12.Page 8, line 142: “strengthens trust built” – Consider rewording | This is corrected line 162 |
| 13.Page 8, lines 136-151: The duration of the participant observations is unclear. Also, please clarify if the ethnographic fieldwork began in January 2020, as stated on Page 7, line 130, or January 2021. | This is corrected and detailed in line 148-151 |
| Results | |
| 14.Page 12, line 248: Please correct the typo | This is corrected line 281 |
| 15.Page 13, line 269: Consider removing “have” | This is corrected line 304 |
| 16.Page 16, lines 325-326: Please consider elaborating “the pain of the treatment process” or add a note that this will be described later in the results | This is corrected line 361 |
| 17.Page 18, lines 364-370: This is an important sub-theme. Isn’t there more information the authors can present about the “many unfounded speculations” regarding the possible side effects of treatment? | This is corrected line 402-403 |
| 18.Page 18, lines 371 – 384: It is important to describe in the methods the different places where CL patients can access treatment in the study district/divisional secretariats. The interchange of terms such as “government hospital clinic”, “treatment centres”, and “teaching hospital” makes the description confusing. | This is corrected in relevant places |
| 19.Page 22, line 474: Is it a “filed note” or a “field note”? | This is corrected line 526 |
| 20.Page 23, line 490: “CL exert an extra burden” – Change to exerts | This is corrected line 527 |
| Discussion |  |
| 21.Page 24, line 511: “people centered care in CL” – Consider rewording to people-centered care for CL. | This is corrected line 548 |
| 22.Page 25, line 519: Could the authors clarify what  “seek health early” means? Consider rewording | This is revised line 559 |
| 23.Page 25, lines 523 – 527: What can be done to reduce the shame and disgust associated with CL at a personal level? | As motioned in Line 582-586 we propose a holistic approach to reduce burden as a whole through providing access to health services, promoting community awareness and education about the disease etc. As the reviewer mentioned about personnel level, we have added the fact as an example in describing personalized care in the discussion section. line 586-590 |
| 24.Page 25, line 532 – Page 26, line 536: The authors point out the need to address the biopsychosocial factors linked to CL. This would involve “providing access to health services, promoting community awareness and education about the disease, and addressing the underlying social determinants of health that contribute to the marginalisation and stigmatisation of affected individuals”. How would this practically be done for people with CL in the study region/rural Sri Lanka? Which players should be involved in tackling each of these elements? Are there examples of how this approach has been used for CL or other NTDs in other countries? | Thank you for this important comment. We have provided examples and explained further on how it is pragmatically possible. Line 592-596 |
| 25.Page 26, line 536: “CL should be improved concerning the biopsychosocial model..” – Did the authors mean considering? | This is corrected line 579 |
| 26.Page 26, lines 537-544: While I whole-heartedly agree about the need of a people-centered approach in dealing with patients with CL, how can healthcare providers understand the “individuals unique needs, preferences, and circumstances” with all the time constraints that accompany their overcrowded clinics? Can the authors provide a recommendation about how this can be practically done? Is this approach only needed at the treatment centres, or are there other interactions that patients with CL have with community health workers or other private or public service providers? | We have revised the discussion section in response to your inquiry. owever, detailed practical implementation of strategies are outside the scope of this specific manuscript which reports about the burden which is the first step of a long process. Guided by the findings of this study, ECLIPSE is working to improve patient care and influence policy changes. As the reviewer recommended, we have added reflections on this aspect in the particular paragraph. (line 578-581and 592-596). |
| 27.Page 26, lines 545-551: Was some degree of socially desirable responding possible, especially with the extensive use of key community members to sensitize and recruit CL participants? Even with the depth of experiences shared by the patients living with CL, is it possible that the true extent of their pain, shame, stigma, and hardships was still not revealed | Thank you for pointing out. We have explained the possibilities in the paragraph on limitations. line 600-607 |
| Also, how did the authors use the ethnographic study information to clarify the PERJ findings? I may have missed the instances where this may have been done in the results and discussion | As mentioned in the methods section (Line 145-151) ethnography was used to culture, beliefs and meanings behind expressions. A comprehensive analysis of all four data components were done (lines 247-264). We have incorporated excerpts from field notes, (line 513-526), PERJ (line 376-379) and post-PERJ interviews (line 391-396) as well.  To enhance clarify we have revised the verbatims to show whether it is from a PERJ or a post-PERJ interview (as PERJ or PERJ-i) (Line 297-298). |
