# Supplementary material for: The psychosocial burden of cutaneous leishmaniasis in rural Sri Lanka: A multi-method qualitative study
Source: PLoS Negl Trop Dis. 2024 Jan 18;18(1):e0011909. doi: 10.1371/journal.pntd.0011909 (PMC10826957; doi:10.1371/journal.pntd.0011909)
Supplement: Attachment — Submitted filename: Response to the reviewers.docx [file pntd.0011909.s005.docx]

Response to reviewers

We thank the reviewers for their comments.

| Reviewer #2 |  |
| --- | --- |
| Methods | |
| The authors' response proficiently clarifies the methodology section, offering a coherent context regarding the adjustments necessitated by the COVID-19 pandemic.  The revisions made to emphasize the process of participant selection and the incorporation of the ECLIPSE Study within the Sri Lankan setting significantly improve the transparency of the methodology employed.  Although the rationale for maintaining the original title is substantiated, it's important to note that the primary contribution of this work predominantly centers around a single qualitative method. The investigators' extensive ethnographic field experience is a valuable asset, enhancing the depth of the study.  The ethical considerations, particularly in the context of the COVID-19 pandemic, are adeptly addressed. | We thank the reviewer for this comment. |
| Results | |
| Delineating how different data sources contributed to the thematic analysis strengthens the transparency and reliability of the findings  The manual analysis process, given the language limitations of software, is insightful. It shows a commitment to ensuring accuracy and relevance in the thematic analysis. | We thank the reviewer for this comment. |
| Editorial and Data Presentation Modifications? | |
| My only minor observation concerns the ethical approval for this study. It appears that the ethical clearance cited (referenced in document 19) pertains to the broader ECLIPSE study and not specifically to this study. Given that the majority of authors are affiliated with Rajarata University, it would be prudent to confirm that there are no issues of dual publication or conflicts of interest related to this overlap. | The ethical approval obtained pertains to the entire ECLIPSE study, under which this specific study was conducted. The methods employed were consistent with those outlined in the approved ethics clearance; therefore, there is no deviation. We also want to highlight that are no concerns regarding dual publications or conflicts of interest, as declared in the competing interest statement. |
| Summary and General Comments | |
| The authors responses to the reviewer comment, reflect deep understanding of both the subject and the research methodologies employed. However, no transcripts or synthesis of those transcripts are fully available as supporting information.   "The PLOS Data policy requires authors to make all data underlying the findings described in their manuscript fully available without restriction, except in cases where the data are legally or ethically restricted (for example, participant privacy is an appropriate restriction)." | The original interview transcripts will not be made fully available in order to safeguard the privacy of the participants.  However, pseudonymized data matrixes containing all data relevant to the study objectives can be made available upon requests made to the corresponding author. |

| Reviewer #3 |  |
| --- | --- |
| The manuscript has been sufficiently revised and meets the standard for publication in PLoS NTD. | We thank the reviewer for this comment. |
